# Prasugrel or ticagrelor relative to clopidogrel in triple-antiplatelet treatment combined with glycoprotein IIb/IIIa inhibitor for patients with STEMI undergoing PCI: a meta-analysis

**DOI:** 10.1186/s12872-020-01403-6

**Published:** 2020-03-12

**Authors:** Zhe Wang, Da-Yan Zhou, Yong Su, Liang-Yi Si, Qiang Xu

**Affiliations:** 1 Department of Cardiology, Chongqing Fifth People’s Hospital, 24 Renji Road, Chongqing, 400062 China; 2 Department of Cardiology, First Hospital Affiliated of Military Medical University (Southwest Hospital), Chongqing, China

**Keywords:** Prasugrel, Ticagrelor, Glycoprotein IIb/IIIa inhibitor, ST segment elevation myocardial infarction, Meta-analysis

## Abstract

**Background:**

For patients with ST-segment elevation myocardial infarction (STEMI) undergoing percutaneous coronary intervention (PCI), the efficacy and safety of novel P2Y_12_ antagonists, including prasugrel or ticagrelor, has not been established relative to that of the clopidogrel-based triple-antiplatelet treatments (TAPTs; in combination with glycoprotein IIb/IIIa inhibitor). The present meta-analysis evaluated the efficacy and safety of prasugrel- or ticagrelor-based TAPTs relative to that of clopidogrel TAPTs in patients with STEMI undergoing PCI.

**Methods:**

The databases PubMed, Embase, and Cochrane’s Library were systematically searched for relevant randomized controlled trials concerning prasugrel or ticagrelor (test) relative to clopidogrel (control). Depending on heterogeneity, studies were pooled with a random effects or a fixed effects model. Outcomes of blood flow after PCI were evaluated, including TIMI (thrombolysis in myocardial infarction), bleeding events, and major adverse cardiovascular events (MACEs).

**Results:**

Seven studies comprising 11,874 patients conformed to the inclusion criteria. The pooled results with the fixed effects model indicated that after PCI patients in the prasugrel or ticagrelor groups were as likely as those treated with clopidogrel to achieve TIMI grade 3 flow or experience bleeding events. However, compared with the control, the test groups had significantly less risk of MACE (OR: 0.81, 95% CI: 0.70*–*0.94, *P* = 0.004), especially at the 1-year follow-up (OR: 0.79, 95% CI: 0.66*–*0.95, *P* = 0.01).

**Conclusions:**

A prasugrel- or ticagrelor-based TAPT may reduce the rate of MACEs, without increasing bleeding in STEMI patients undergoing PCI. However, due to the limited RCT studies and variations in study weight, results of this meta-analysis should be confirmed in a large RCT with adequate sample size and follow-up duration.

## Background

For patients with ST-segment elevation myocardial infarction (STEMI) undergoing percutaneous coronary intervention (PCI), the efficacy and safety of novel P2Y_12_ antagonists, relative to clopidogrel, remains unclear when either is combined with aspirin and glycoprotein IIb/IIIa inhibitor (GPI). Some randomized controlled trials (RCTs) have compared the newer P2Y_12_ antagonists and clopidogrel-based triple-antiplatelet treatment (TAPT) for these patients [1–7]. However, the results have been inconsistent, perhaps due to variations in sample sizes.

The present meta-analysis evaluated the efficacy and safety of the P2Y_12_ antagonists prasugrel or ticagrelor, relative to that of clopidogrel-based TAPTs, in patients with STEMI undergoing PCI. In particular, the associated rates of acute and long-term adverse events were investigated, including blood flow after PCI, bleeding events, and major adverse cardiovascular events (MACEs).

## Methods

### Data source and search strategy

This meta-analysis was performed in accordance with the guidelines of PRISMA (Preferred Reporting Items For Systematic Reviews And Meta-Analyses Group For Randomized Controlled Trials) [8] and the Cochrane’s Handbook of Systematic Review and Meta-analysis [9]. Electronic databases, including PubMed, EMBASE, and CENTRAL (Cochrane Central Register of Controlled Trials) were systematically searched for relevant studies, using combinations of the following terms: “AMI”, “acute coronary syndrome”, or “myocardial infarction”; “abciximab”, “tirofiban”, “eptifibatide”, “platelet glycoprotein IIbIIIa”, or glycoprotein IIbIIIa; “ticagrelor”; and “prasugrel”. In addition, the reference lists of the retrieved articles and reviews were manually scanned for relevant studies. No restrictions for language were applied when performing the database search.

### Inclusion and exclusion criteria

Studies were included if they met the following criteria: designed as an RCT; patients with STEMI undergoing PCI; patients were randomly assigned to a loading dose of either ticagrelor or prasugrel, or a loading dose of clopidogrel, in combination with periprocedural GPIs; and follow-up ≥30 days. In addition, all RCTs reported at least one of the following outcomes, or these data could be calculated: achievement of thrombolysis in myocardial infarction (TIMI) blood flow grade 3 after PCI; MACEs; and bleeding events. Major bleeding was based on the TIMI criteria (http: //www.timi.org/). Conference abstracts and duplicated RCTs were excluded.

### Outcome definition

Major bleeding complications were defined according to the TIMI hemorrhage classification in the TIMI (thrombolysis in myocardial infarction) [2] and FABOLUS PRO (facilitation through Aggrastat by dropping or shortening infusion line in patients with STEMI compared to or on top of prasugrel given at loading dose) [4] trials. The definitions of major bleeding complications were study-specific in Liu et al. [7] and the PLATO (platelet inhibition and patient outcomes) trial [3]. Outcomes were based on the longest follow-up available. The diagnosis of STEMI and definitions of MACE were those of the original articles. MACEs included all-cause mortality, non-fatal myocardial infarction, non-fatal stroke, and target vascular revascularization. Follow-up data were collected in the 7 trials at 30 days and at 12 months.

### Data extraction and quality assessment

Two authors (XQ and DZ) independently performed the database search, data extraction, and quality assessment. Disagreements were resolved by consensus. Data were extracted that regarded study design, patient characteristics, intervention treatments, and outcomes. The quality of the RCTs was assessed using Cochrane’s tool for the risk of biases [9], which included the following: sequence generation, concealment of allocation, blinding, incomplete outcome data, selective outcome reporting, and potential sources of other bias.

### Statistical analyses

For continuous outcomes, the weighted mean difference and 95% confidence interval (CI) were used to describe the pooled results. For categorized outcomes, the odds ratio (OR) and 95% CI were calculated. Heterogeneity among the included RCTs was analyzed by Cochrane’s Q test [9]. The *I*^2^ statistic was calculated, with values < 25, 25 to 50%, and > 50% indicating low, moderate, and high heterogeneity, respectively [10].

A fixed effects model was applied to pool the results of the RCTs, if no significant heterogeneity was detected (*P* > 0.1, *I*^2^ ≤ 50%); otherwise, a randomized effects model was applied. Pre-specified subgroup analyses were performed according to the severity of bleeding or follow-up duration to explore the potential sources of heterogeneity [11]. Publication bias was determined based on the symmetry of the funnel plot [12]. Statistical analyses were performed with RevMan Software (Version 5.3, Cochrane Collaboration Network for Meta-analyses, UK).

## Results

### Database search

**Fig. 1:**
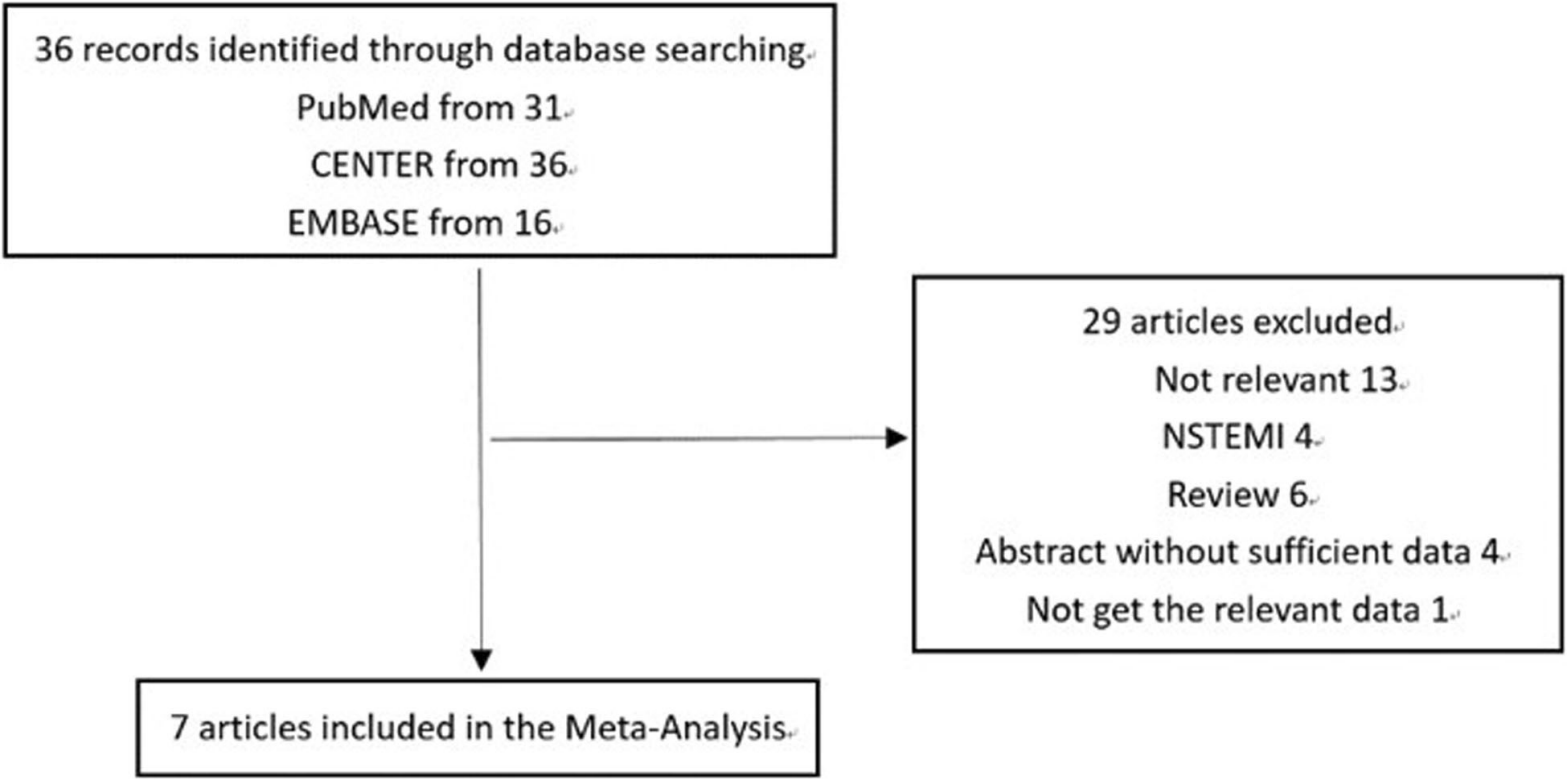
Flowchart of database search

Initially, 36 potentially relevant articles were retrieved from the literature search (Fig. 1). After screening the titles and abstracts, 29 articles were excluded, mostly due to irrelevancy, the patients did not have STEMI, or an abstract did not publish sufficient data. Another study was excluded because related outcome data were not reported. Therefore, 7 studies were finally included in the current meta-analysis [1–7].

### Study characteristics

In the final analysis, data from 7 studies comprising 11,874 patients were included, and all studies used a GPI(s) to varying degrees (4–100%). The analysis was restricted to patients who had received a GPI with STEMI undergoing PCI. Most of the GPI administration was by tirofiban bolus with or without post-bolus tirofiban infusion. Some studies did not indicate the method of GPI administration. In the PLOT [3] study, the proportion of patients using GPI was 26.6%. We only analyzed the data of this subgroup of the patients with STEMI using GPI. In the PLOT study, about 45% of the patients used proton pump inhibitors, but no specific outcome data of the patients with STEMI using GPI were available for further analysis. Other studies did not describe the use of proton pump inhibitors.

In the study by Liu et al. [7], the radial access accounted for 92.4%, while in the study by Christ et al. [5], the radial access accounted for 7%. In the study by Schulz et al. [6], the femoral access accounted for 99.8%. Other inclusion studies did not state the PCI approach.

All of the included RCTs were published during the years 2009 to 2015 (Table 1). The sample sizes of the included RCTs varied from 56 to 18,624. The percentages of men varied from 42 to 88%, and mean ages ranged from 51.9 to 80 years. Considerable percentages of patients had the conventional risk factors or comorbidities of coronary heart disease, including: hypertension (24.5–67.3%), diabetes (9.7–100%), and smoking (57–68.8%). The follow-up period ranged from one month to one year.

**Table 1:** Characteristics of the included RCT studies^a^

	n ^b^	GPI, %	Age, y	Male	Hypertension	DM	Dyslipidemia	Smoking	FU
Brener, et al., 2014 [1]	452	50	57.6 ± 10.4/62.7 ± 12.7	79.4/71	24.5/35.0	9.7/12.1	16.8/15.2	68.8/58.3	1 y
Christ, et al., 2013 [5]	56	100	57 ± 11/67 ± 13	74/88	48/77	28/39	84/94	60/58	1 mo
PLATO, 2009 [3]	7026 ^c^	26	62/62 ^d^	71.6/71.7	65.8/65.1	24.9/25.1	46.6/46.7	N/A	1 y
Michelle, 2009 [2]	3534 ^c^	54.5	60/60 ^d^	76.7/75	61.2/61.7	22.7/22.3	56.4/57.2	N/A	1 mo
BRAVE, 2014 [6]	548	4	51.9–71.7/52.9–71.5	76/79	66/64	17/15	57/51	57/67	1 mo
FABOLUS PRO, 2012 [4]	100	48	66 ± 11/67 ± 10	69/78	63/79	19/21	50/64	N/A	1 mo
Yang, 2015 [13]	158	66.5	60.6 ± 7.7	42%	67.3	100	N/A	28.85–38.85	1 mo

^a^ Reported as percentage, unless noted otherwise; ^b^ subjects; ^c^ STEMI; ^d^ median age, risks factor are from the TAPT cohorts

Abbreviations: *DM* diabetes mellitus; *FU* follow-up

### TIMI grade 3 flow after PCI

**Fig. 2:**
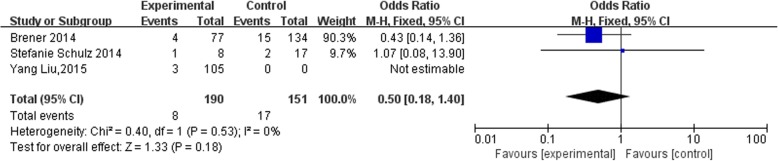
Forest plot for the meta-analysis of TIMI grade 3 blood flow achievement in patients assigned to prasugrel or ticagrelor as compared with clopidogrel on the basis of GPI

Three of the studies [1, 6, 7] evaluated the efficacy of prasugrel or ticagrelor combined with GPI, relative to clopidogrel combined with GPI, with regard to achieving TIMI grade 3 flow after PCI (Fig. 2). No significant heterogeneity was detected among the included RCTs (*P* value for Cochrane’s Q test = 0.53, *I*^2^ = 0%). The pooled results with a fixed effects model indicated that all the treatments were comparable with regard to achieving TIMI grade 3 flow after PCI (prasugrel or ticagrelor cf. clopidogrel, OR: 0.50, 95% CI: 0.18*–*1.40, *P* = 0.18).

### Risk of bleeding

**Fig. 3:**
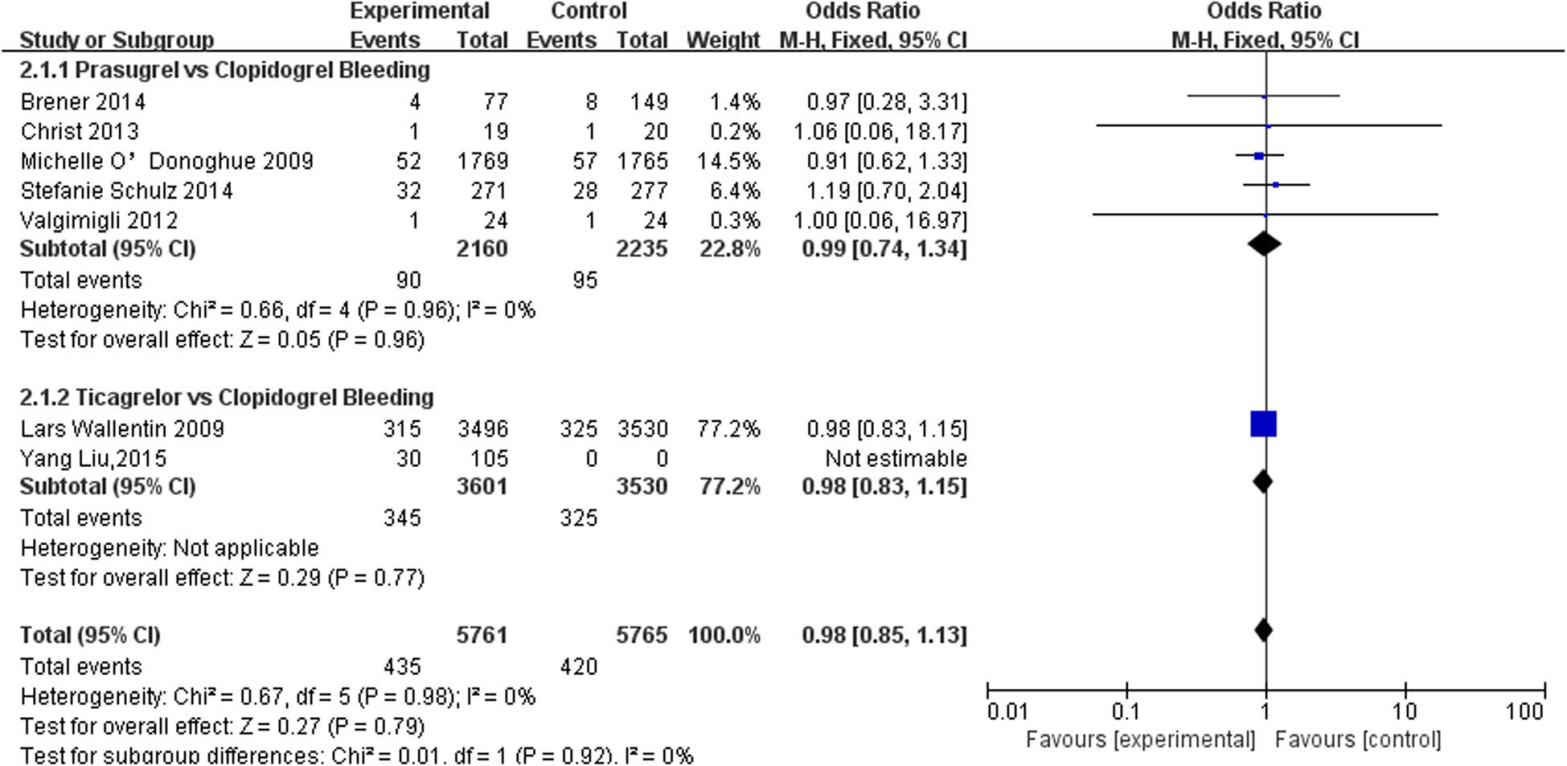
Forest plot for the meta-analysis of risk of bleeding in patients assigned to prasugrel or ticagrelor as compared with clopidogrel on the basis of GPI

Six of the included studies reported the rates of bleeding [2–7] (Fig. 3). There was no significant heterogeneity detected among these RCTs (*P* value for Cochrane’s Q test = 0.96, *I*^2^ = 0%). The pooled results with a fixed effects model indicated that the rates of bleeding events, as defined by the TIMI standards, were comparable (prasugrel or ticagrelor with GPI cf. clopidogrel with GPI, OR: 0.98, 95% CI: 0.85*–*1.13, *P* = 0.79).

### Mace

**Fig. 4:**
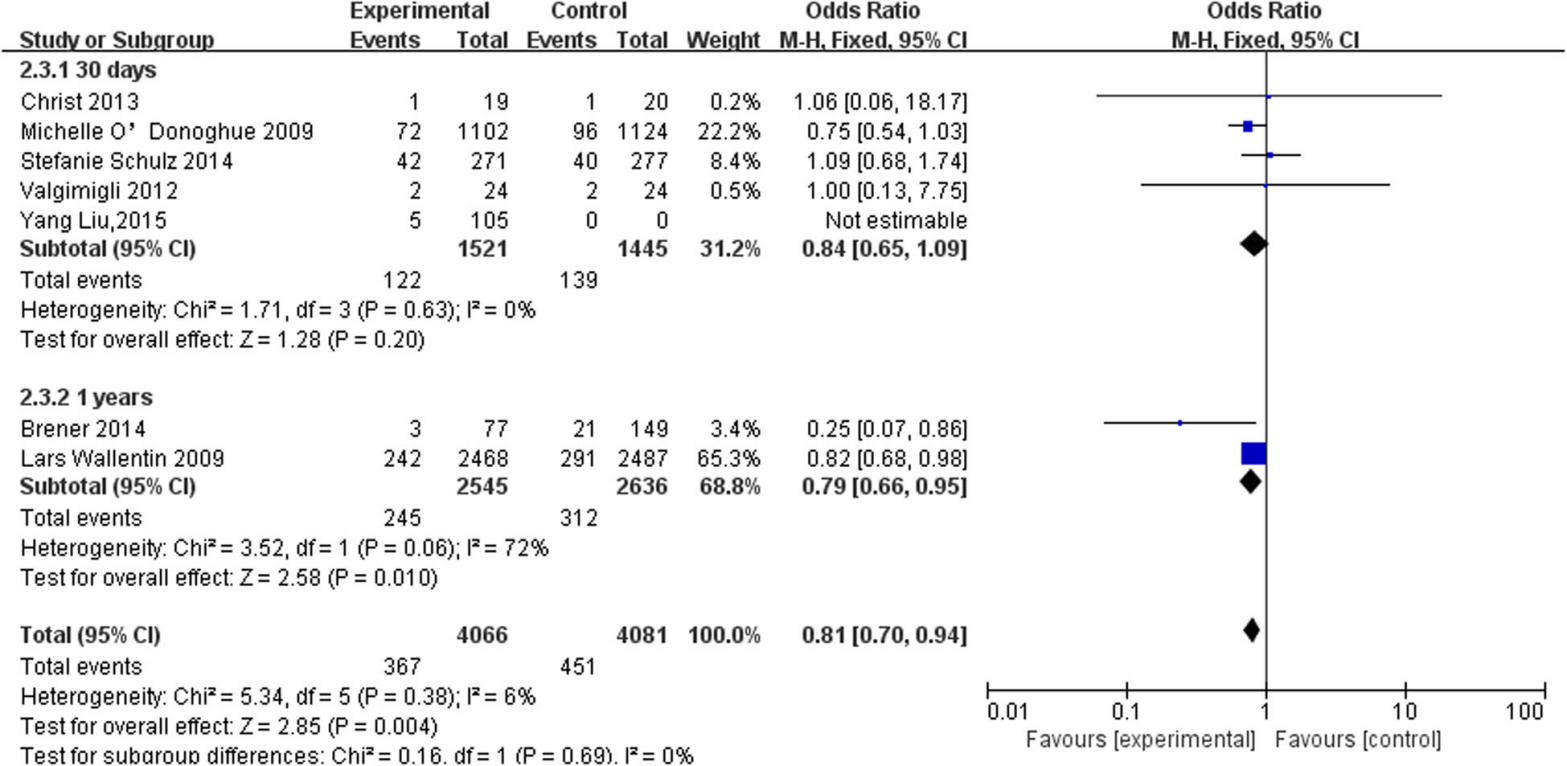
Forest plot for the subgroup analysis of risk of MACE in patients assigned to prasugrel or ticagrelor as compared with clopidogrel on the basis of GPI: stratified according to the follow-up duration

All of the 7 included studies reported the rates of MACEs (Fig. 4) [1–7]. For the FABOLUS PRO [4] trial, MACE data was clearly recorded in the experimental and control groups (mortality, 2; reinfarction and urgent target vessel revascularization, nil). For the study by Liu et al. [7], within 30 days there were 5, 3, and 2 cases of MACE, mortality, and reinfarction, respectively. For the study by Christ et al. [5], death occurred in 5 cases, without stent thrombosis. The PLOT trial and other studies were without individualized data.

The pooled results with a fixed effects model indicated that use of prasugrel or ticagrelor, with GPI, was associated with a significantly lower rate of MACE compared with clopidogrel with GPI (OR: 0.81, 95% CI: 0.70*–*0.94, *P* = 0.004).

Subsequent analyses stratified by duration of follow-up showed that the rates of MACEs within 30 days did not differ among the groups (prasugrel or ticagrelor with GPI cf. clopidogrel with GPI, OR: 0.84, 95% CI: 0.65*–*1.09, *P* = 0.20). The rates of MACEs within 1 year were significantly lower in the groups treated with prasugrel or ticagrelor compared with that of clopidogrel (OR: 0.79, 95% CI: 0.66*–*0.95, *P* = 0.01). However, the difference between rates of MACEs at 30 days and 1 year were not significant (*P* = 0.69).

### Publication bias

**Fig. 5:**
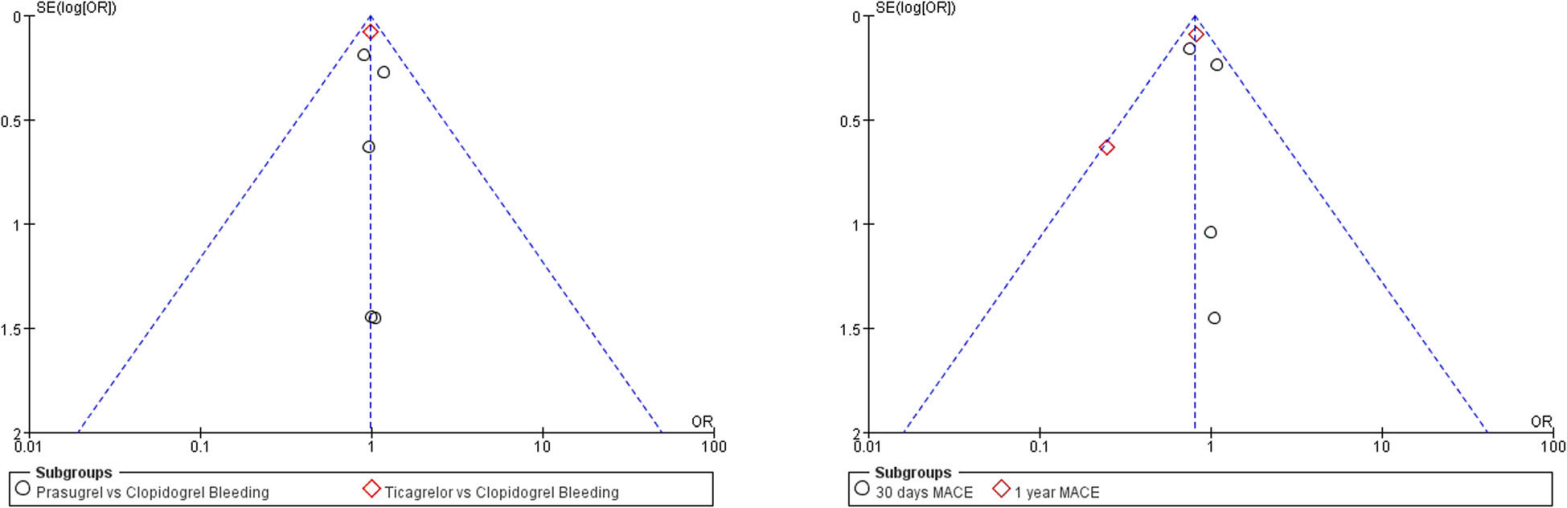
Funnel plots for the meta-analysis of bleeding and MACE in patients assigned to prasugrel or ticagrelor as compared with clopidogrel on the basis of GPI

Visual inspection of funnel plots did not support the presence of significant publication bias in the meta-analysis (Fig. 5). Quantitative analyses of publication bias with Egger’s tests were not possible due to the limited number of studies.

## Discussion

By pooling the results of all available RCTs, we found that a prasugrel- or ticagrelor-based TAPT did not significantly affect the achievement of TIMI grade 3 flow after PCI, or rates of bleeding events, compared with the clopidogrel-based TAPT in patients with STEMI undergoing primary PCI. However, during follow-up significantly less risk of MACE was associated with the prasugrel- or ticagrelor-based TAPT compared with the clopidogrel-based TAPT. Results of subgroup analyses confirmed that the observed benefits of prasugrel- or ticagrelor-based TAPT on clinical outcomes were mainly due to the reduced incidence of 1-year MACE in these groups. Taken together, these results suggest that, for patients with STEMI undergoing PCI, TAPT with prasugrel or ticagrelor in combination with aspirin and GPI may significantly reduce the risk of MACE without increasing the risk of bleeding events, compared with the classic clopidogrel-based TAPT. Our results support the use of the P2Y_12_ antiplatelet medications ticagrelor or prasugrel over that of clopidogrel-based TAPT for STEMI patients undergoing PCI.

The comparative efficacy and safety of the newer P2Y_12_ antiplatelet medications and clopidogrel for patients with coronary heart disease have been evaluated previously in a few meta-analyses. An early meta-analysis comprising 12 RCTs suggested that oral P2Y_12_ inhibitors significantly reduced the rate of ischemic events (OR = 0.85) without significantly increasing major bleeding in patients with coronary heart disease, and the risk/benefit ratio was particularly favorable for STEMI patients (OR = 0.77) [13]. For patients with non-ST segment elevation acute coronary syndrome, results of a subsequent meta-analysis of 4 RCTs indicated that a novel P2Y_12_ antiplatelet was associated with a significantly reduced rate of MACE compared with clopidogrel (risk ratio [RR] = 0.87), but the incidences of major and minor bleeding events were significantly higher (RR = 1.27, 1.20) [14]. For patients with STEMI, results of 3 meta-analyses of RCTs consistently indicated that prasugrel and ticagrelor were more efficacious than clopidogrel for reducing the risk of MACE, although the rates of bleeding events were similar [15–17].

To our best knowledge, none of the above meta-analyses considered the safety and efficacy of novel P2Y_12_ antiplatelet medication-based TAPT. Since use of GPI has proved effective to reduce the number of acute coronary no-reflow and stent thrombosis events, the efficacy of novel P2Y_12_ antiplatelet medication-based TAPTs deserves evaluation in high-risk patients with STEMI undergoing PCI. Moreover, use of novel P2Y_12_ antiplatelet medication-based TAPTs may expose patients to higher risk of bleeding events, and therefore the safety of the above regimens compared with conventional clopidogrel-based TAPT is of particular importance. Our results indicate that for patients with STEMI undergoing PCI, TAPT with prasugrel or ticagrelor in combination with aspirin and GPI may significantly reduce the risk of MACE without increasing the risk of bleeding events, as compared with the classic clopidogrel-based TAPT. This suggests that use of novel P2Y_12_ antiplatelet medication-based TAPTs may be favorable for high-risk patients with STEMI undergoing PCI. These beneficial effects of novel P2Y_12_ antiplatelet medication-based TAPTs on clinical outcomes in patients with STEMI undergoing PCI may be explained by the potential pharmacological advantages of prasugrel or ticagrelor that have been confirmed in previous studies [18, 19]. However, whether other mechanisms are involved should be investigated.

Regarding the relative efficacies of prasugrel and ticagrelor-based antiplatelet regimens for patients with STEMI undergoing PCI, results of previous findings may provide some evidence. Serebruany et al. [17] found that prasugrel, but not ticagrelor, offers a significant 30-day mortality benefit over clopidogrel in PCI-treated STEMI patients. This was confirmed by a subsequent meta-analysis, which showed that for STEMI patients undergoing PCI, prasugrel was superior to ticagrelor, particularly in conjunction with bivalirudin and drug-eluting stents [15]. Direct evidence was presented in a recently published meta-analysis of head-to-head RCTs, in which prasugrel appeared equivalent or superior to ticagrelor for patients with acute coronary syndrome undergoing PCI at the 30-day follow-up [20]. However, whether prasugrel based-TAPTs are superior to ticagrelor based-TAPTs in STEMI patients undergoing PCI remains to be determined.

Our study has limitations which should be considered when interpreting the results. Firstly, the number of studies included in the meta-analysis was small. In this study, STEMI patients with atrial fibrillation were not included because no relevant data was reported. Therefore, results of the current analysis could not be extrapolated to this particular population. The conclusions should be confirmed in a large RCT with adequate sample size and follow-up duration. Secondly, the patients’ characteristics, coronary lesions, PCI features, and doses of perioperative medications varied among the RCTs, and we did not have access to the individual patient data. This made it difficult to perform subgroup analyses to evaluate whether differences in these study characteristics could significantly affect the outcome. Finally, although visual inspection did not support significant publication bias of the meta-analysis, quantitative analyses could not be conducted due to the limited number of studies.

## Conclusions

A TAPT with prasugrel or ticagrelor in combination with aspirin and GPI may significantly reduce the risk of MACEs without increasing the risk of bleeding events in patients with STEMI undergoing primary PCI, compared with the classic clopidogrel-based TAPT. These findings warrant confirmation in a large RCT with adequate sample size and follow-up duration.

## Data Availability

The datasets generated and analyzed during the current study are available from the corresponding author on reasonable request.

## Abbreviations

AHA: American Heart Association
CAD: Coronary artery disease
CI: Confidence interval
MACEs: Major adverse cardiovascular events
PCI: Percutaneous coronary intervention
STEMI: ST-segment elevation myocardial infarction
TAPT: Clopidogrel-based triple-antiplatelet treatments
